# Predictive modeling of immune escape and antigenic grouping of SARS-CoV-2 variants

**DOI:** 10.1128/jvi.00225-26

**Published:** 2026-04-27

**Authors:** Arshan Nasir, Diana Lee, Laura E. Avena, Daniela Montes Berrueta, Tessa Speidel, Kai Wu, Yadunanda Budigi, Andrea Carfi, Guillaume B. E. Stewart-Jones, Darin Edwards

**Affiliations:** 1 Moderna, Inc. 380888 , Cambridge, Massachusetts, USA; The Ohio State University, Columbus, Ohio, USA

**Keywords:** COVID-19, deep mutational scanning, immune escape, SARS-CoV-2 variants, predictive modeling, bioinformatics

## Abstract

The ongoing adaptive evolution of Severe acute respiratory syndrome coronavirus-2 (SARS-CoV-2) is characterized by the continued emergence of variants with increased transmissibility and the ability to escape infection- and/or vaccine-induced immunity. This sustained antigenic evolution has necessitated updates to COVID-19 vaccine compositions to better match circulating viral variants. To optimize protection against emerging variants, a reliable means of predicting the immune escape of novel variants is needed to enable at-risk preparation of new vaccine strain compositions. Herein, we describe the development and applications of a quantitative risk calculator that predicts relative immune escape of SARS-CoV-2 variants using a statistical modeling framework. The approach integrates large-scale, experimentally derived spike-antibody epitope and escape maps with serum neutralization data generated using pseudotyped viruses and clinical sera. By aggregating site-level escape information into a strain-level metric, the calculator enables the grouping of antigenically related SARS-CoV-2 variants to guide strain selection for at-risk vaccine design and preparation, in anticipation of seasonal strain change recommendations by global public health agencies and the WHO. Here, we demonstrate the utility of this framework through retrospective and prospective strain selection exercises for the XBB.1.5-, JN.1/KP.2-, and LP.8.1-adapted mRNA-1273 COVID-19 vaccines during the 2023–2026 seasons, respectively. In all cases, model predictions were largely supported by clinical immunogenicity data and aligned with subsequent recommendations by global public health agencies.

**IMPORTANCE:**

We present a framework to estimate the relative immune escape potential of emerging variants by integrating previously published experimental epitope-level escape data with serum neutralization measurements. By consolidating mutation-level effects into a strain-level metric, this approach enables classification of antigenically similar variants. Retrospective and prospective applications demonstrate that model-based assessments are consistent with observed immunogenicity data. This framework provides a practical tool to support preparedness efforts by informing at-risk vaccine development activities in advance of seasonal strain selection guidance.

## INTRODUCTION

Severe acute respiratory syndrome coronavirus-2 (SARS-CoV-2) continues to undergo sustained antigenic evolution, resulting in the repeated emergence of variants with potentially altered transmissibility and immune escape properties (1, 2). Since its initial emergence in 2019, inter-host viral evolution has been largely shaped by a balance between selection for improved receptor engagement and evasion of humoral immunity, with the surface spike glycoprotein (S) receptor-binding domain (RBD) serving as the primary target of neutralizing antibodies (3, 4). In parallel, prolonged intra-host viral evolution, particularly in immunocompromised individuals, has likely contributed to the repeated emergence of highly divergent “long-branch” or saltation variants, which periodically spill over into the broader population (5). These combined inter- and intra-host viral evolutionary processes have driven successive global strain replacement events since 2020, including the transitions from the ancestral Wuhan-Hu-1 strain to Delta, Omicron BA.1, BA.2, BA.5, XBB, BA.2.86/JN.1, and most recently, BA.3.2 variants. Timely identification and characterization of emerging high-risk immune-evasive SARS-CoV-2 variants are thus vital for proactive adjustments to approved vaccines to optimize continued efficacy against evolving strains (6).

Amino acid substitutions in the S RBD protein play a central role in immune escape by directly altering antibody binding sites or indirectly modifying spike conformation and accessibility (3, 4). Deep mutational scanning (DMS) and large-scale antibody mapping studies have previously revealed that a relatively constrained set of RBD residues disproportionately contributes to neutralization escape across diverse monoclonal antibodies (mAbs) and polyclonal sera (7–10). These findings have enabled increasingly precise molecular descriptions of antigenic change, yet translating mutation-level information into strain-level predictions of immune escape remains a major challenge. Current experimental approaches for assessing immune escape, including pseudovirus (PsV) or live virus neutralization assays (11), are resource intensive and inherently retrospective. While these assays remain essential for validation, they are poorly suited for rapid response to the expanding landscape of emerging variants detected through routine global genomic surveillance. Consequently, there is a growing need for complementary approaches ranging from sequence-based computational models (12, 13) to experimentally grounded frameworks (7) to prospectively estimate immune escape risk.

Here, we describe the development and application of a risk calculator that predicts relative loss of neutralization for emerging SARS-CoV-2 variants using an antibody binding-based framework. By integrating large-scale antibody escape maps derived from previously published DMS studies (7–10) with statistical modeling trained on clinical serum neutralization data, the risk calculator provides a rapid and quantitative *in silico* estimate of the potential immune escape of emerging variants, enabling grouping of antigenically related strains and prioritization of strains for at-risk vaccine development, including assessment of preclinical and clinical immunogenicity. Here we demonstrate the utility of this strategy in at-risk strain selection and preparation of updated mRNA-1273 COVID-19 vaccines in the 2023–2026 seasons and show how these predictions closely aligned with subsequent clinical immunogenicity data.

## MATERIALS AND METHODS

### Clinical trial and serum neutralization activity

Serum neutralization activity was measured using a vesicular stomatitis virus (VSV)-based PsV assay (14) to generate experimental data for training the risk calculator. Specifically, Day 29 sera (4 weeks after the fourth dose) from healthy adult participants (*n* = 20) enrolled in the Phase 2/3 Part H study (NCT04927065) were collected (15). These sera were tested for neutralization activity against a panel of 18 SARS-CoV-2 pseudotyped variants that were epidemiologically relevant at the time of model training (April 2023; see Table 1). Study protocols and results have been reported previously (15). Briefly, eligible participants in the Part H study had received a primary series (two doses) of mRNA-1273 (the original vaccine targeting the ancestral Wuhan-Hu-1 strain), followed by a third booster dose of mRNA-1273 and a fourth booster dose of mRNA-1273.222 (the 2022–2023 bivalent vaccine formulation targeting the ancestral Wuhan-Hu-1 and BA.4/BA.5 strains) (15). Among the 20 participants, 10 had a history of prior infection.

**TABLE 1:** Neutralization data for pseudotyped SARS-CoV-2 variants against sera from the mRNA-1273.222 Phase 2/3 Part H clinical trial*^a^*^,*b*^

Variant	Neutralizing antibody GMT (ID_50_)	Log_10_ titer	ABI
BA.1	3,214	3.507	0.0977
BA.5	3,328	3.5222	0
BA.2.75	1,679	3.2251	0.0771
BA.2.75.2	428	2.6314	0.2077
BN.1	574	2.7589	0.3037
CH.1.1	186	2.2695	0.2477
DS.1	190	2.2788	0.3762
XBB.1.5	254	2.4048	0.3073
BA.2.3.20	972	2.9877	0.2439
BJ.1	824	2.9159	0.2665
CM.8.1.1	558	2.7466	0.3264
XAY.2	1,185	3.0737	0.095
XBF	211	2.3243	0.2714
BQ.1.1	519	2.7152	0.1716
BH.1	1,231	3.0903	0.166
BF.3.1	1,637	3.214	0.0432
XBB.1	213	2.3284	0.3073
XBB.1.16	254	2.4048	0.3129

^*a*^ GMT, geometric mean titer; ID_50_, 50% inhibitory dose; S, spike glycoprotein.

^*b*^ Calculated ABIs are listed for filtered mutations by comparing to the BA.5 S protein (mRNA-1273.222 bivalent vaccine composition for 2022–2023).

### Antibody binding data and escape profiles

Antibody S epitopes and escape profiles were retrieved from previously published data sets from the Cao and Bloom laboratories (7–10). These data sets collectively describe antibody and S contact positions for >4,000 mAbs determined via DMS, along with individual mutations and sites likely to cause loss of antibody binding (and by extension, immune escape) (7–10). Using these data, we derived an antibody binding index (ABI) to summarize the predicted antibody escape potential of a given spike variant. The ABI aggregates site-specific escape effects across a large antibody repertoire and is conceptually similar to escape metrics previously described by Bloom and colleagues (7) while extending the framework to variant-level comparisons and vaccine-relevant mutations.

### Derivation of the ABI

**Fig 1:**
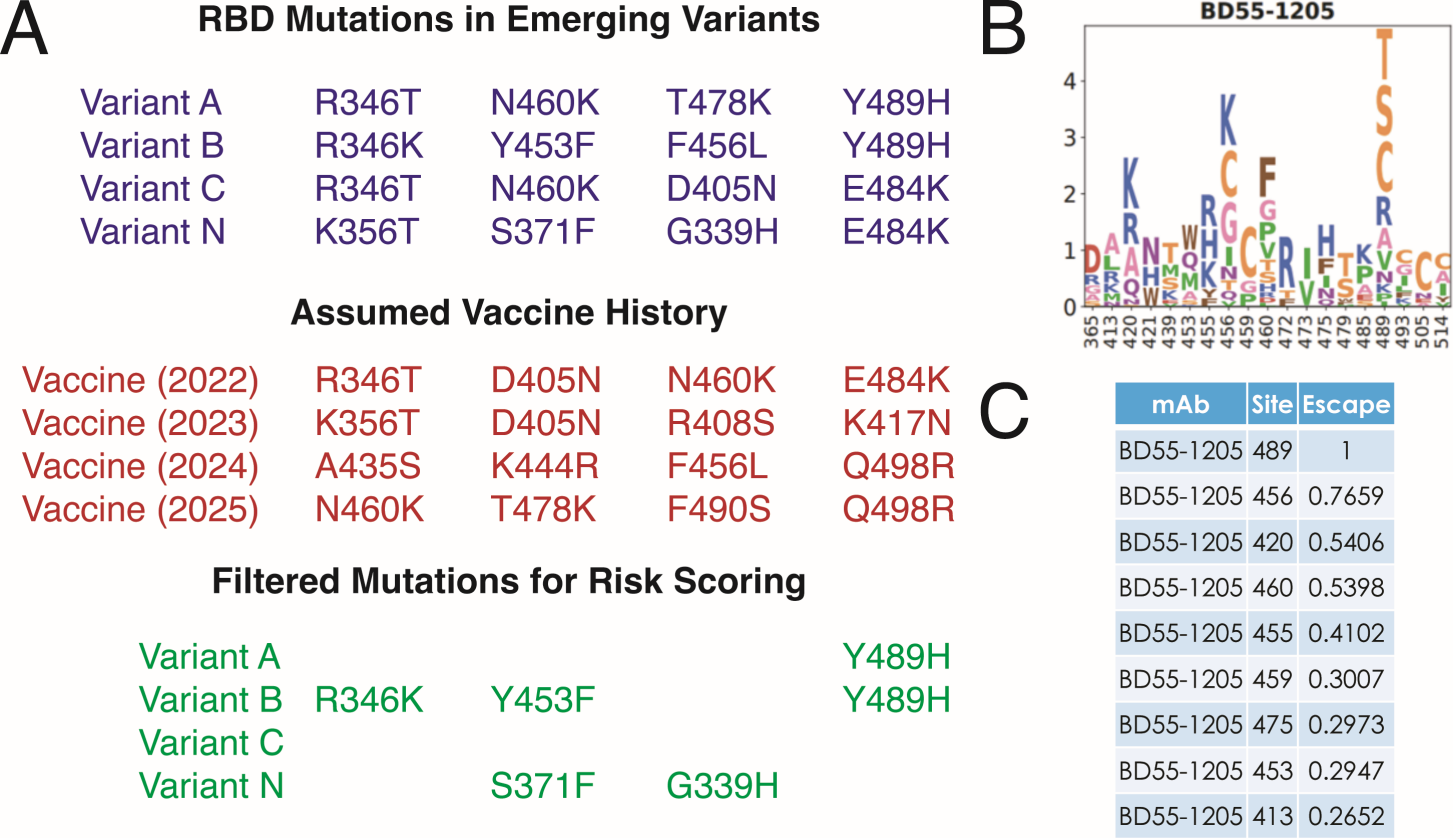
Conceptual framework for derivation of the antibody binding index (ABI). (**A**) Schematic illustration of RBD mutations detected in example SARS-CoV-2 variants, shown relative to an assumed vaccine antigen history. For each variant, only newly introduced mutations, including amino acid changes occurring at previously mutated sites, are retained for ABI calculation (“filtered mutations”), while mutations already present in prior vaccine strains are excluded. (**B**) Representative antibody escape map for the monoclonal antibody BD55-1205 (generously provided by Yunlong Cao, May 2023), illustrating site-specific escape contributions across the RBD as quantified by previously published DMS experiments (8, 9). Letter height reflects the relative magnitude of escape conferred by substitutions at each site. (**C**) Example subset of site-level escape scores for BD55-1205, highlighting that certain positions (e.g., residue 489) confer strong escape, whereas others contribute partial escape. Retrieved from https://jbloomlab.github.io/SARS2-RBD-escape-calc/ (accessed May 2023). For a given antibody, escape scores corresponding to all filtered mutations present in a variant are summed and capped at a maximum value of 1 to reflect saturation of antibody escape. This procedure is repeated across all antibodies in the panel (>4,000 mAbs), and the ABI is calculated as the mean capped escape across antibodies, yielding a normalized, variant-level measure of predicted escape.

For a given variant 𝑣, let 𝑀_𝑣_ denote the set of “filtered” amino acid mutations in the S RBD that are newly introduced relative to prior vaccine strains, including new amino acid changes occurring at previously mutated sites (Fig. 1A). For each antibody 𝑗 in the data set (𝑁 > 4,000), we retrieved the site-specific escape scores 𝐸_𝑗,𝑖_ associated with each mutation 𝑖 ∈𝑀_𝑣_ that falls within the predicted epitope for antibody 𝑗, as determined via the DMS (7–10). The 𝐸_𝑗,𝑖_ scores were obtained from the Bloom laboratory SARS-CoV-2 RBD escape calculator (https://jbloomlab.github.io/SARS2-RBD-escape-calc/, accessed May 2023) (7), which provides curated access to published DMS data sets. Each 𝐸_𝑗,𝑖_ represents the experimentally measured fractional escape of antibody 𝑗 due to mutation 𝑖, scaled between 0 (no measurable reduction in binding) and 1 (strongest site of escape) (7). For each antibody, escape contributions across all mutations were summed and capped at one to represent complete loss of binding, consistent with prior work (7). Formally, the ABI for variant 𝑣 is defined as:


$$ABI\;(v)=\frac{1}{N}\sum_{j=1}^{N}min\left(1,\;\sum_{i\in M_{v}}E_{j,i}\right).$$

The ABI thus represents the mean fractional loss of predicted antibody binding across the antibody panel due to mutations present in variant 𝑣. ABI values are bounded between 0 and 1, with higher values indicating broader predicted escape across. Antibodies were weighted equally in the calculation, irrespective of neutralization potency. Because escape scores are aggregated at the site level and assumed to contribute additively, the ABI does not explicitly model amino acid-specific interactions or epistatic effects between mutations within a given genetic background.

### Risk score calibration and statistical analysis

To translate the ABI values into strain-level risk scores reflecting predicted immune escape, ABI values were calibrated against experimentally measured serum neutralization titers. For each of the 18 SARS-CoV-2 variants included in the training data set (Table 1), the ABI values were paired with corresponding pseudovirus neutralization titers derived from Day 29 sera collected in the Phase 2/3 Part H clinical study (NCT04927065) (15). Neutralization titers were log_10_-transformed prior to analysis to stabilize variance and reflect the multiplicative nature of antibody-mediated neutralization. A linear regression model was fitted to describe the relationship between ABI (predictor) and log-transformed neutralization titers (dependent variable). Model parameters were estimated using ordinary least squares, and goodness of fit was assessed using the coefficient of determination (*R*²) and residual diagnostics. Model significance was evaluated using an *F* statistic (16). Model assumptions were validated formally using the Shapiro-Wilk and Breusch-Pagan tests for normality and equal variances, respectively (17, 18). This calibration step established a quantitative mapping between predicted antibody binding disruption and observed reductions in serum neutralization. Strain-level risk scores were then derived by converting ABI values for emerging variants into predicted fold reductions in neutralization relative to a reference vaccine strain. Risk scores were expressed as fold changes to facilitate comparison with experimentally measured neutralization data and to enable intuitive interpretation in the context of vaccine performance. Antigenic grouping was performed based on the similarity of ABI values. The same model was used to extrapolate neutralization scores for newer variants against the more recent vaccines, including mRNA-1273.815 (the 2023–2024 monovalent vaccine formulation targeting the XBB.1.5 variant), mRNA-1273.167 (the 2024–2025 monovalent vaccine formulation targeting the JN.1 variant), mRNA-1273.712 (the 2024–2025 monovalent vaccine formulation targeting the KP.2 variant), and mRNA-1273.251 (the 2025–2026 monovalent vaccine formulation targeting the LP.8.1 variant).

### Sequence data sources and variant annotation

SARS-CoV-2 genome sequences were obtained from public repositories, including GISAID (19) and GenBank (20), and analyzed locally. Nextclade algorithm (21) was used for viral genome alignment, quality control checks, mutation calling, and clade assignment. S mutations were called relative to the ancestral Wuhan-Hu-1 strain (GenBank Accession ID: MN908947) using in-house Python (version 3.12.2) scripts.

### Recombinant VSV-PsV assay

Codon-optimized full-length S genes for the 18 pseudotyped variants (Table 1) were cloned into a pCAGGS vector. To generate VSVΔG-based SARS-CoV-2 PsV, BHK-21/WI-2 cells were transfected with the S expression plasmid and infected by VSVΔG-firefly-luciferase as described previously (22–24). Vero E6 cells (ATCC, CRL-1586) were used as target cells for the neutralization assay and were maintained in Dulbecco’s Modified Eagle Medium (DMEM) supplemented with 10% fetal bovine serum (FBS). To perform the neutralization assay, human serum samples were heat-inactivated for 30 minutes at 56°C, and serial dilutions were made in DMEM supplemented with 10% FBS. The diluted serum samples or culture medium (serving as a virus-only control) were mixed with VSVΔG-based SARS-CoV-2 PsV and were incubated at 37°C for 45 minutes. The inoculum virus or virus–serum mix was subsequently used to infect Vero E6 cells for 18 hours at 37°C. At 18 hours after infection, an equal volume of One-Glo reagent (Promega, E6120) was added to the culture medium for readout using a BMG PHERastar-FSX plate reader. The percentage of neutralization was calculated based on relative light units of the virus control and was subsequently analyzed using a four-parameter logistic curve (GraphPad Prism version 10.2.1).

## RESULTS

### Quantitative relationship between ABI and serum neutralization

**Fig 2:**
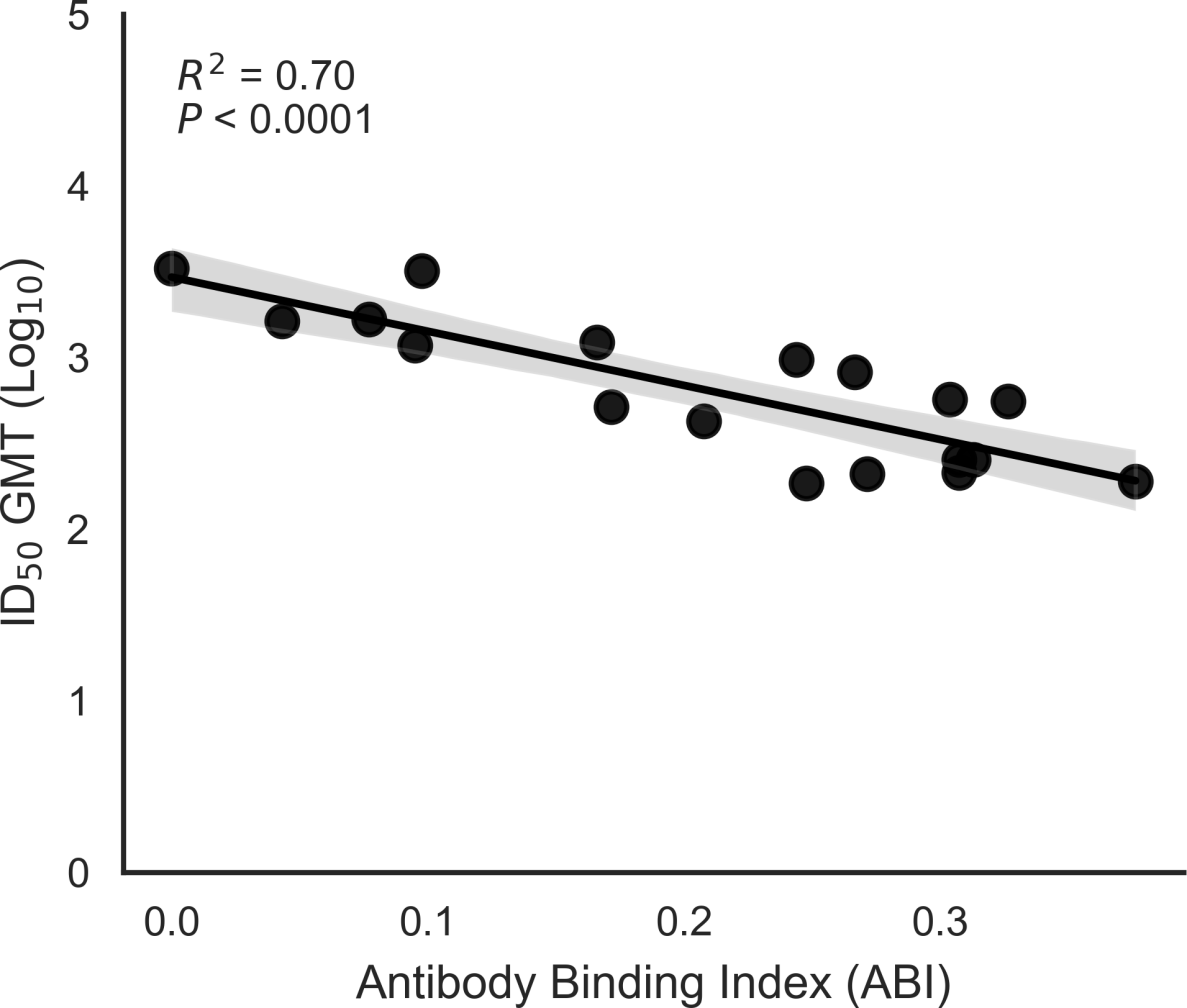
Statistical relationship between the ABI (*x*-axis) and serum neutralization for SARS-CoV-2 variants evaluated against clinical sera from the mRNA-1273.222 Phase 2/3 Part H study (*y*-axis). The solid line represents the fitted linear regression relating ABI to log_10_ ID_50_ geometric mean titers (GMTs), with the shaded region indicating the 95% CI. The ABI explains approximately 70% of the observed variance in neutralization titers (*R*² ≈ 0.70, *P* < 10^−4^). Regression diagnostics indicated no strong deviation from normality or homoscedasticity (Shapiro–Wilk and Breusch–Pagan tests, *P* > 0.3). Ab, antibody; ID_50_, 50% inhibitory dose.

We first evaluated the relationship between the ABI and experimentally measured serum neutralization to assess whether the ABI provided a quantitative proxy for immune escape at the strain level. ABI values were calculated for 18 SARS-CoV-2 variants and compared against corresponding pseudovirus neutralization titers measured using Day 29 sera from participants in the Phase 2/3 Part H clinical study (NCT04927065, Table 1). Across variants, ABI values showed a strong inverse relationship with log_10_-transformed neutralization titers (Fig. 2). Variants with higher ABI values, indicating greater predicted disruption of antibody binding across the monoclonal antibody panel, consistently exhibited lower serum neutralization titers, whereas variants with lower ABI values retained higher neutralization. Overall, the linear regression analysis demonstrated that ABI explained a substantial proportion of the observed variance in neutralization titers [*R*^2^ = 0.7; *F* value (1, 16) = 38.03; *P* < 0.0001], indicating that mutation-level antibody escape information can be aggregated into a single quantitative metric that reflects functional immune escape. Importantly, this relationship was monotonic across the range of variants tested, spanning strains that were well matched to the vaccine antigen as well as those exhibiting substantial immune escape. The consistency of this relationship supported the use of ABI as a calibrated, strain-level measure of predicted immune escape and provided the foundation for subsequent risk score assignment and antigenic grouping of emerging variants.

### Risk calculator enables antigenic grouping of SARS-CoV-2 variants and predicts cross-neutralization from approved vaccines

A key utility of the risk calculator is its potential to calculate risk scores for emerging variants against licensed SARS-CoV-2 vaccine compositions, as well as new variant vaccine candidates that may be under development. The input to the risk assessment model is the S amino acid sequence (or a list of RBD mutations) for a given SARS-CoV-2 variant (Fig. 1A). The output is a numerical value (called risk score) that describes the expected relative drop in neutralization for the variant from previously approved vaccines. The risk scoring and ranking enable rapid identification and prioritization of immune-evasive variants based solely on *in silico* analysis for further epidemiologic monitoring and potential commercial development, if needed.

**Fig 3:**
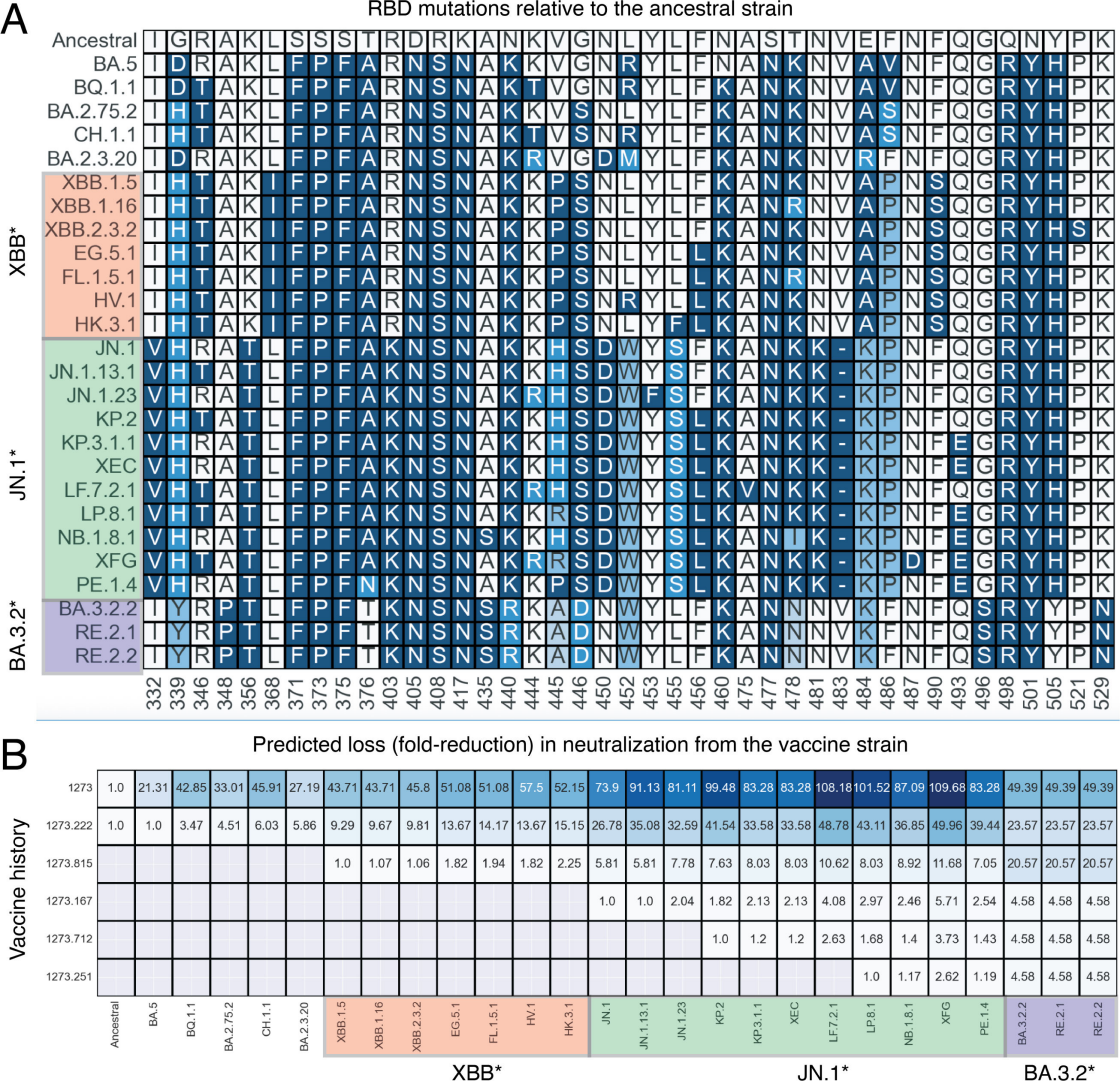
Antigenic grouping of variants and predicted immune escape by vaccination history. (**A**) S RBD mutations in the epidemiologically relevant SARS-CoV-2 variants in the prior and current seasons. The *x*-axis indicates the amino acid position relative to the ancestral (Wuhan-Hu-1) strain. (**B**) The matrix lists the predicted immune escape for the variants against different immunization backgrounds. The values inside the matrix are the predicted fold drop in the ID_50_ neutralizing antibody GMTs on the vaccine strain, derived or extrapolated from the statistical model described in Fig. 2. The risk scores against any new vaccine assume prior exposure to all the previous vaccine variants either via immunization or infection. The baseline risk scores for the JN.1 lineage from the XBB.1.5 vaccine were adjusted using neutralization data from reference 24. NB.1.8.1 evolved from XDV, a recombinant with a JN.1-like S, and included in the JN.1 grouping from an escape perspective. GMT; geometric mean titer; ID_50_, 50% inhibitory dose; RBD, receptor-binding domain; S, spike glycoprotein; mRNA-1273, the original vaccine formulation targeting the ancestral Wuhan-Hu-1 strain; mRNA-1273.222, the 2022–2023 bivalent vaccine formulation targeting the ancestral Wuhan-Hu-1 and BA.4/BA.5 strains; mRNA-1273.815, the 2023–2024 monovalent vaccine formulation targeting the XBB.1.5 variant; mRNA-1273.167, the 2024–2025 monovalent vaccine formulation targeting the JN.1 variant; mRNA-1273.712, the 2024–2025 monovalent vaccine formulation targeting the KP.2 variant; and mRNA-1273.251, the 2025–2026 monovalent vaccine formulation targeting the LP.8.1 variant. Asterisk indicates the same lineage.

The RBD mutations and predicted risk scores for a range of variants since the ancestral Wuhan-Hu-1 to currently circulating XFG, NB.1.8.1, and BA.3.2.2/RE.2.2 variants against a panel of previously or currently authorized/approved vaccines, including mRNA-1273, mRNA-1273.222, mRNA-1273.815, mRNA-1273.167, mRNA-1273.712, and mRNA-1273.251 vaccines, are shown in Fig. 3. In the 2023–2024 season, the risk calculator indicated that XBB.1.5 had a greater risk score (9.29) against the mRNA-1273.222 (the 2022–2023 bivalent vaccine formulation targeting the ancestral Wuhan-Hu-1 and BA.4/BA.5 strains) vaccine authorized at that time, compared with other co-circulating strains such as BQ.1.1, BA.2.75.2, CH.1.1, and BA.2.3.20, which had substantially lower risk scores (3.47–6.03, Fig. 3B). The higher risk score associated with XBB.1.5 was likely due to the presence of several new and convergent RBD mutations in the XBB.1.5 S RBD, including G339H, R346T, L368I, V445P, G446S, N460K, F486P, and F490S (Fig. 3A), that likely conferred a higher degree of immune escape and fitness to XBB.1.5 compared with other strains circulating at that time. The risk calculator also enabled antigenic grouping of variants within the XBB lineage. For example, XBB-derived sublineages XBB.1.16, XBB.2.3.2, EG.5.1, FL.1.5.1, HV.1, and HK.3.1, which emerged either during the XBB.1.5 infection wave or soon after, had risk scores comparable to that of XBB.1.5 (1.06- to 2.25-fold difference, Fig. 3B). Collectively, this information derived from the risk analysis suggested that an XBB.1.5 vaccine was likely to cross-neutralize all those strains and was likely the most suitable candidate for a vaccine update.

In late 2023, JN.1 (a descendant of BA.2.86) emerged and quickly displaced the XBB subvariants as the globally predominant strain. JN.1 was characterized by ~60 S mutations relative to the ancestral Wuhan-Hu-1 strain and >30 mutations compared with the XBB.1.5 strain (25, 26), which was included in the mRNA-1273.815 vaccine for the 2023–2024 season. Some notable mutations in the JN.1 S included K356T, which introduced a new N-linked glycosylation site in the RBD; N460K, which likely improved ACE2 binding affinity; L455S, which likely improved the transmission capabilities of JN.1; and several additional NTD and RBD substitutions, insertions, and deletions likely to improve immune escape (25–27). The risk calculator initially overestiamted the risk score for JN.1 against the mRNA-1273.815 vaccine (see Discussion). Initial neutralization testing revealed a 5.8-fold drop in nAb titers to JN.1 compared with the XBB.1.5 strain in the sera from mRNA-1273.815-boosted individuals (24). We therefore set 5.8 as the baseline risk score for predictive modeling of JN.1 subvariants against the mRNA-1273.815 vaccine. In 2024-25, all JN.1 subvariants including JN.1.13.1, JN.1.23, KP.2, and KP.3.1.1. had risk scores comparable to that of JN.1 (1- to 2.13-fold difference), with the highest risk score for KP.3.1.1 (2.13 against the JN.1 vaccine, Fig. 3B). The risk scores were improved when considering a KP.2 vaccine composition, suggesting that initial JN.1 and KP.2 were well suited as candidate vaccine strains for the 2024–2025 season.

Following the selection of JN.1 and KP.2-based vaccine compositions for use throughout the 2024–2025 COVID vaccination period (28, 29), continued antigenic drift and recombination gave rise to successive dominant JN.1 subvariants, including KP.3.1.1 (alias for JN.1.11.1.3.1.1), XEC (a recombinant of two JN.1 subvariants), NB.1.8.1 (evolved from XDV, which itself was a complex recombinant with a JN.1-like S), LF.7.2.1 (alias for JN.1.16.1.7.2.1), and LP.8.1 (alias for JN.1.11.1.1.1.3.8.1). Early antigenic profiling demonstrated that LF.7.2.1 was more immune evasive than XEC but exhibited weaker ACE2 engagement, whereas LP.8.1 showed immune escape comparable to XEC with improved ACE2 binding, consistent with its increased transmission and subsequent rise in multiple countries (30). Both LP.8.1 and LF.7.2.1 differed from JN.1 at 8-9 S positions, including RBD substitutions such as K444R and A475V in LF.7.2.1 and V445R in LP.8.1 (Fig. 3A). Predictive modeling indicated that vaccines based on mRNA-1273.167 (JN.1) or mRNA-1273.712 (KP.2) would be expected to cross-neutralize these newer JN.1 strains (Fig. 3B). Although LF.7.2.1 exhibited relatively higher predicted immune escape of 4.08 against the JN.1 vaccine and 2.63 against the KP.2 vaccine (Fig. 3B), similar to prior published studies (30), LF.7.2.1 was ultimately outcompeted by fitter variants such as LP.8.1, which was later recommended as the preferred vaccine composition in the United States (31) and as a suitable alternative by the WHO in May 2025 (32).

More recently, XFG has become dominant in the United States (33) and parts of Europe, while NB.1.8.1 remains prevalent in several Asian countries, and BA.3.2 has expanded rapidly in Europe and parts of Australia. Travel-associated cases of BA.3.2 have been detected elsewhere, including the United States (34, 35). In addition, PE.1.4, a subvariant of KP.3.1.1, has been detected and has shown growth in some parts of Australia. XFG is a recombinant virus derived from JN.1 subvariants, including LF.7 and LP.8.1.2, with a recombination breakpoint within the RBD (36). As of February 2026, XFG accounts for approximately 65% of sequenced cases in the United States (33). In contrast, BA.3.2 represents a long-branch variant characterized by >60 spike substitutions relative to both the ancestral and JN.1 strains, likely arising from prolonged evolution in an immunocompromised host over several years (37). BA.3.2 has diversified into BA.3.2.1 and BA.3.2.2, with BA.3.2.2 becoming more globally prevalent and further splitting into RE.2.1 (expanding in Australia) and RE.2.2 (expanding in Europe with travel-associated cases elsewhere). Notably, the consensus S sequences for BA.3.2.1, RE.2.1, and RE.2.2 share identical RBD sequences (Fig. 3A). Our predictive modeling assigns a risk score of 2.62 for XFG and 4.58 for BA.3.2.2/RE.2.2 against the mRNA-1273.251 (LP.8.1) vaccine (Fig. 3B), indicating moderate immune-escape potential, especially for BA.3.2.2/RE.2.2, which should continue to be monitored for further antigenic evolution and transmission potential.

### Clinical cross-neutralization data validate predictive risk modeling across the 2023–2024 and 2024–2025 seasons

To assess whether the ABI-based risk framework accurately or closely predicted immune escape observed in humans, we compared model-derived risk scores with clinical serum neutralization data from the 2023–2024 (XBB.1.5) and 2024–2025 (JN.1/KP.2 adapted) mRNA vaccine evaluations (38, 39). Across both seasons, the relative magnitude and ranking of neutralization escape measured experimentally largely mirrored the *in silico* predictions generated by the risk calculator prior to or contemporaneous with strain selection.

For the 2023–2024 season, the XBB.1.5-based vaccine (mRNA-1273.815) was evaluated as a fifth dose in U.S. adults previously vaccinated with mRNA-1273, boosted with mRNA-1273, and subsequently boosted with mRNA-1273.222 (Part J, NCT04927065; *n* = 50) (38). At Day 29 post-vaccination, robust nAb responses were observed across all tested variants. Consistent with the risk model predictions, the nAb fold increase from pre-booster levels was higher against the XBB.1.5, XBB.1.16, EG.5.1, BA.2.86, and JN.1 variants (10.4- to 19.0-fold) compared with the D614G, BA.4/BA.5, and BQ.1.1 variants (4.4- to 7.3-fold). The nAb titers at Day 29 after mRNA-1273.815 vaccination were lowest against EG.5.1, BA.2.86, and JN.1 and were reduced by 2.5- to 5.8-fold compared with those elicited against the target variant, XBB.1.5. Overall, the direction and relative magnitude of experimentally measured neutralization reductions aligned closely with model-derived risk estimates.

Similarly, clinical evaluations of the 2024–2025 vaccine compositions further validated the predictive framework. Both the JN.1-encoding vaccine (mRNA-1273.167) and the KP.2-encoding vaccine (mRNA-1273.712) were administered as booster doses, with Day 29 titers assessed against vaccine-matched variants (JN.1 and KP.2) and non-matched JN.1-lineage subvariants, including KP.3.1.1, XEC, and LP.8.1 (39). Both vaccines induced strong increases in nAb titers against matched variants (GMFR, 11.6-11.7) and demonstrated broad cross-neutralization of related JN.1-derived strains (39). Consistent with model predictions, cross-neutralization was reduced relative to the matched reference for all non-matched subvariants. For example, the measured fold reduction was ~2.0 for XEC against the JN.1-adapted vaccine and 1.2 against the KP.2-adapted vaccine, which nearly perfectly matched with the modeled risk estimates (2.13 vs JN.1 and 1.2 vs KP.2, Fig. 3B). Similarly, the modeled risk scores for LP.8.1 (2.97 vs JN.1 vaccine and 1.68 vs KP.2 vaccine) also closely matched measured reduction (2.35 and 1.15, respectively). KP.3.1.1 exhibited slight deviations with the measured escapes of 3.42 and 1.71 against the JN.1 and KP.2-adapted vaccines, respectively, compared to 2.13 and 1.2 outputted by the risk calculator (Fig. 3B).

Collectively, these clinical data sets spanning two consecutive seasons demonstrated that the risk calculator generally anticipated both the presence and relative magnitude of neutralization escape observed in human sera. These findings support the utility of the framework as a prospective decision-support tool for antigenic grouping, strain prioritization, and evaluation of cross-neutralization potential prior to or in parallel with experimental testing.

### Application of the risk calculator to real-time surveillance of emerging variants

We next applied the ABI-based risk assessment framework to currently circulating or recently emerging SARS-CoV-2 variants identified through ongoing global genomic surveillance. This analysis focused on variants that rose following the 2025-2026 vaccine strain selection (LP.8.1 adapted), including XFG, NB.1.8.1, and BA.3.2 and their respective sublineages. Variants or recombinants derived from the JN.1 S backbone, including XFG and NB.1.8.1, were assigned relatively low to moderate risk scores (2.62 for XFG and 1.17 for NB.1.8.1) when evaluated against the LP.8.1-based vaccine (mRNA-1273.251, Fig. 3B). Both XFG and NB.1.8.1 were thus predicted to retain substantial cross-neutralization by LP.8.1-based vaccines. For both variants, we observed improvements in the risk score when moving from JN.1 to KP.2 to LP.8.1, indicating the benefit of updating the vaccine composition to LP.8.1 earlier in 2025. In contrast, BA.3.2 exhibited moderately higher relative risk scores of 4.58, irrespective of the JN.1 member vaccine (Fig. 3B). BA.3.2 represents a long-branch lineage that diverged substantially from both ancestral and JN.1-derived strains, likely arising from prolonged intra-host evolution (37). Importantly, despite continued geographic expansion and sublineage diversification within BA.3.2, major BA.3.2 subvariants (RE.2.2 and RE.2.1) shared similar risk scores, enabling lineage-level prioritization rather than repeated evaluation of individual subvariants. Notably, these prospective risk estimates were consistent with subsequent clinical immunogenicity assessments of LP.8.1-containing vaccines, in which lower neutralization was observed against BA.3.2 relative to JN.1-related variants (40). This concordance between modeled predictions and measured neutralization further supports the practical utility of the framework in real-time surveillance settings. Together, these results demonstrate that the risk calculator can be applied prospectively to newly detected variants to rapidly distinguish incremental antigenic drift within established lineages from more substantial antigenic shifts arising through recombination or long-branch evolution. While the actual risk scores may differ between the model and experimentally-derived risk scores, the relative ranking of variants is expected to remain relatively more robust. This capability supports timely prioritization of variants for experimental evaluation and at-risk vaccine preparation as the SARS-CoV-2 variant landscape continues to evolve.

## DISCUSSION

Here we describe the utility of a risk calculator to predict the relative immune escape of emerging SARS-CoV-2 variants and to prioritize at-risk preparation of strains for annual and/or off-cycle vaccine updates, if needed. The risk calculator provides a quick assessment of potential immune escape of emerging variants and enables antigenic grouping of variants to predict cross-neutralization. Such data are informative both for internal decision-making and for at-risk preparation of vaccine constructs, if later requested by the global public health agencies. This is especially useful when there is co-circulation of multiple strains with comparable growth rates (e.g., in case of incremental or stepwise convergent evolution of virus) but also helpful when more mutated strains emerge either via reverse zoonosis or chronic spillovers or recombination with some important differences, as discussed below. While the risk calculator provides a quantitative and scalable approach for estimating immune escape, several limitations should be considered when interpreting the predicted risk scores.

First, the framework is expected to perform most accurately for variants that arise through stepwise antigenic drift within established lineages, where new mutations accumulate incrementally over time. The current implementation assumes that site-level escape effects are additive and independent and therefore does not explicitly model epistatic interactions between mutations within a given genetic background. In such cases, exemplified by XBB- and JN.1-derived subvariants, the model effectively captures relative differences in immune escape and supports antigenic grouping and prioritization. In contrast, variants arising through long-branch evolutionary events may initially be over- or underestimated by the model. These variants (e.g., JN.1 and BA.3.2) often accumulate large numbers of spike mutations over short evolutionary intervals and may experience distinct immune pressures compared with variants circulating broadly in the population (5, 37). In addition, context-dependent effects of specific amino acid S substitutions may differ across variant backbones, which are not explicitly modeled in the current framework. In these scenarios, periodic recalibration, adjustments, or retraining of the model using updated clinical sera and emerging variant data may be needed to maintain predictive accuracy.

Second, the risk calculator is strictly trained on pandemic sera and antibodies (the majority targeting the RBD) and included all the antibodies included in the published DMS studies by Cao’s laboratory at the time of model development (2023). The ABI framework currently weights all antibodies equally and does not incorporate experimentally measured differences in neutralization potency. In the future iterations of the model, we plan to integrate potency-weighted estimates to better approximate population-level neutralization landscapes. Another future update will be to expand the model to include NTD-targeting antibodies. NTD mutations can have conformational effects on the RBD, including altering RBD up/down positioning, and may indirectly improve virus immune evasion, as highlighted by the past success of KP.3.1.1 and XEC variants, which were both characterized by new NTD glycosylation sites (41). Such structural or conformational effects outside the RBD are not explicitly captured by the current model. Similarly, a recent study showed that infants primed/infected with only the XBB strain had serum neutralization activity mostly targeted towards the NTD, in contrast to RBD-targeting neutralization activity in adults primed with the ancestral Wuhan-Hu-1 strain (42). Such heterogeneities exist at the population level but are not currently modeled by the risk calculator. Moreover, a large fraction of the global population is now likely exposed to multiple SARS-CoV-2 strains, either via repeated infections or vaccinations or both (43). The order of infections and vaccinations also differs at the population level; infants are now primed only with the most recently approved vaccines, which may impact the specificity and target of the serum neutralization response. Presently, the calculator does not distinguish between immunity acquired either via infection or vaccination or both. Similarly, the immune response to variants also involves additional components such as T-cell responses and host-related factors (44, 45), which are not currently modeled. We aim to implement these more complex features in future updates of the model.

More than 4,000 antibodies from previously published studies were incorporated in the calculation of the ABI and used to train the risk calculator (7–10). This rich data set includes antibodies with diverse binding properties, although the majority bind preferentially to the ancestral Wuhan-Hu-1 strain and were isolated earlier in the pandemic. Therefore, the antibody landscape represented in the model may not fully reflect the evolving antibody repertoire in populations repeatedly exposed to Omicron-lineage viruses. Notably, not all antibodies in the data set are neutralizing, as epitopes were defined using DMS and escape scores for individual mutations were aggregated at the site level. As a result, the model does not explicitly capture amino acid-specific effects beyond the aggregated site-level escape scores, nor does it account for epistatic interactions that may arise in different variant genetic backgrounds. In addition, the current risk assessment model was trained using serum neutralization data from a Phase 2/3 Part H clinical study involving mRNA-1273.222-boosted sera (15) and is applied to predict immune escape against more recently authorized vaccines. Ideally, it will be important to update the risk assessment model annually, coinciding with each new vaccine update or the emergence of a new saltation/long-branch variant. However, logistical constraints, including the availability of sufficient clinical sera, antibodies, and pseudotyped variants for model training and validation, have limited the feasibility of routine annual updates. Recent sequence-based methods, including ESM-2 (12) and EVEscape (13), represent important advances in using deep learning and protein language models to estimate immune escape or fitness effects of viral mutations directly from sequence data. While such approaches offer speed and scalability, experimentally anchored frameworks such as the one presented here provide direct linkage to measured antibody escape and clinical neutralization responses. Future work that integrates sequence-based predictions with experimentally derived escape landscapes may further enhance early immune escape risk assessment.

Despite these stated limitations, we believe that the current framework remains appropriate for making relative statements about immune escape. The virus continues to evolve antigenically by acquiring new S mutations that can affect antibody binding and thereby neutralization; therefore, the inferred statistical relationship between antibody binding and neutralization loss is likely to remain robust through subsequent iterations of vaccines and variants, as shown by cross-validations with clinical data in the 2023–2026 seasons. While the absolute risk scores may fluctuate, as additional variants and antibodies are added to the training data set of the model, the relative rankings and relationships between variants are likely to remain robust. Consistent with this, the risk calculator has maintained strong predictive performance against newer variants and vaccine compositions, despite being originally trained on clinical data generated in 2022–2023. As SARS-CoV-2 continues to evolve, rapid prioritization of strains for potential vaccine updates remains essential. The risk calculator enables a quick quantitative assessment of immune escape for emerging subvariants to help prioritize strains for experimental assessments from a much larger pool of virus genetic diversity and co-circulating variants. Risk scores generated by the model, coupled with growth trends of variants, provide valuable information about the ongoing adaptive evolution of SARS-CoV-2. In the future, we aim to continue to perform regular updates to the risk calculator by integrating the latest pandemic sera from the sponsor’s ongoing clinical trials and DMS profiles of new antibody S complexes, as and when they become publicly available. Moreover, the developed tools can be adapted to provide similar assessments against other adaptive pathogens such as seasonal influenza viruses, efforts that could enable better strain matching and improved influenza vaccine effectiveness.

### Conclusion

We describe our response to the ongoing adaptive evolution of SARS-CoV-2 based on routine and active genomic surveillance combined with bioinformatics-based risk assessment. Our risk calculator allows us to identify candidate vaccine strains within a condensed time window to support seasonal updates, if later requested by the health agencies. We continue to monitor SARS-CoV-2 evolution and assess and report on the impact of new variants against the latest vaccine formulations, including the assessment of variants that become globally dominant and those variants that show potential for immune escape from the current season’s vaccine composition.

## Data Availability

Access to participant-level data presented in this article and supporting clinical documents with external researchers who provide methodologically sound scientific proposals will be available upon reasonable request for products or indications that have been approved by regulators in the relevant markets and subject to review from 24 months after study completion. Such requests can be made to Moderna, Inc., 325 Binney St Cambridge, MA 02139 (data_sharing@modernatx.com) and to the corresponding authors (Arshan.Nasir@modernatx.com> or <darin.edwards@modernatx.com>. A material transfer and/or data access agreement with the sponsor will be required for accessing shared data. All other relevant data are presented in the paper. The protocol is available online at ClinicalTrials.gov (NCT04927065). The example genomic sequence data and metadata for variants reported in this study can be accessed via EPI_SET_250127qx. To view the contributors of each individual sequence with details such as accession number, virus name, collection date, originating lab, submitting lab, and the list of authors, visit 10.55876/gis8.250127qx. Data to reproduce the statistical model described in this study are provided in Table 1. ABI calculation follows previously published work (7–10).
